# In Favor of a Banquet

**Published:** 1889-09

**Authors:** Benj. W. Bristow

**Affiliations:** Flatonia, Tex.


### IN FAVOR OF A BANQUET.

FLATONIA, TEX., July 19, 1889.

*Editor Daniel's Texas Medical Journal:*

I see that some of our friends are expressing themselves earnestly, and, as I think, a little too "previously" upon the subject of banquet or no banquet at our next meeting at Fort Worth. From any standpoint this seems to me in very bad form;—we go to Fort Worth in the interest of the medical profession; and for what we receive, let us be truly thankful. That is outside of the medical features; but let us not say to our brethren in the Fort, what they shall do for our entertainment. As for myself I am frank to acknowledge that, like Prince Hal, "I do, by my troth, often remember the poor creature, small beer"—much more "a good sherris sack,"—and if the Fort Worth doctors and the good people there should determine to give us such a spread as we had at San Antonio, why, I flatter myself that in the matter of eating and drinking, I shall do no mean part, and if there be those who do not enjoy banquets—well, attendance is not compulsory,--and as to the, to me, rather cheap morality, presented by some of our friends, let them remember that "notwithstanding they are virtuous, there shall still be cakes and ale."

Wishing the JOURNAL and yourself personally every success,

I am, very truly, yours,

BENJ. W. BRISTOW, M. D.

